# Subclinical Hypothyroidism after Radioiodine Exposure: Ukrainian–American Cohort Study of Thyroid Cancer and Other Thyroid Diseases after the Chornobyl Accident (1998–2000)

**DOI:** 10.1289/ehp.0800184

**Published:** 2008-12-15

**Authors:** Evgenia Ostroumova, Alina Brenner, Valery Oliynyk, Robert McConnell, Jacob Robbins, Galina Terekhova, Lydia Zablotska, Ilya Likhtarev, Andre Bouville, Viktor Shpak, Valentin Markov, Ihor Masnyk, Elaine Ron, Mykola Tronko, Maureen Hatch

**Affiliations:** 1 Radiation Epidemiology Branch, Division of Cancer Epidemiology and Genetics National Cancer Institute, National Institutes of Health, Department of Health and Human Services, Bethesda, Maryland, USA;; 2 Institute of Endocrinology and Metabolism, Kyiv, Ukraine;; 3 Department of Medicine, The Thyroid Clinic, College of Physicians and Surgeons, Columbia University, New York, New York, USA;; 4 Clinical Endocrinology Branch, National Institute of Diabetes and Digestive and Kidney Diseases, National Institutes of Health, Department of Health and Human Services, Bethesda, Maryland, USA;; 5 Department of Epidemiology, Mailman School of Public Health, Columbia University, New York, New York, USA;; 6 Scientific Center for Radiation Medicine, Academy of Medical Science, Kyiv, Ukraine

**Keywords:** Chernobyl nuclear accident, Chornobyl, dose–response relationship, hypothyroidism, ionizing radiation

## Abstract

**Background:**

Hypothyroidism is the most common thyroid abnormality in patients treated with high doses of iodine-131 (^131^I). Data on risk of hypothyroidism from low to moderate ^131^I thyroid doses are limited and inconsistent.

**Objective:**

This study was conducted to quantify the risk of hypothyroidism prevalence in relation to ^131^I doses received because of the Chornobyl accident.

**Methods:**

This is a cross-sectional (1998–2000) screening study of thyroid diseases in a cohort of 11,853 individuals < 18 years of age at the time of the accident, with individual thyroid radioactivity measurements taken within 2 months of the accident. We measured thyroid-stimulating hormone (TSH), free thyroxine, and antibodies to thyroid peroxidase (ATPO) in serum.

**Results:**

Mean age at examination of the analysis cohort was 21.6 years (range, 12.2–32.5 years), with 49% females. Mean ^131^I thyroid dose was 0.79 Gy (range, 0–40.7 Gy). There were 719 cases with hypothyroidism (TSH > 4 mIU/L), including 14 with overt hypothyroidism. We found a significant, small association between ^131^I thyroid doses and prevalent hypothyroidism, with the excess odds ratio (EOR) per gray of 0.10 (95% confidence interval, 0.03–0.21). EOR per gray was higher in individuals with ATPO ≤ 60 U/mL compared with individuals with ATPO > 60 U/mL (*p* < 0.001).

**Conclusions:**

This is the first study to find a significant relationship between prevalence of hypothyroidism and individual ^131^I thyroid doses due to environmental exposure. The radiation increase in hypothyroidism was small (10% per Gy) and limited largely to subclinical hypothyroidism. Prospective data are needed to evaluate the dynamics of radiation-related hypothyroidism and clarify the role of antithyroid antibodies.

The 26 April 1986 accident at the Chornobyl (Chernobyl) nuclear power plant contaminated large areas of northern Ukraine as well as parts of Belarus and the Russian Federation. The environmental fallout included radionuclides of iodine, primarily iodine-131 (^131^I), which concentrates in the thyroid gland (United Nations Scientific Committee on the Effects of Atomic Radiation 2000). The scientific evidence accumulated since the accident points clearly to a substantial increase in thyroid cancer among those exposed as children (Cardis et al. 2005; Ivanov et al. 2006; Jacob et al. 1999; Likhtarov et al. 2006; Tronko et al. 2006b). However, research into possible effects of exposure on thyroid function has been limited, and the results have been inconsistent, largely because of issues in study design and lack of individual dose estimates (Eheman et al. 2003; Goldsmith et al. 1999; Kasatkina et al. 1997; Pacini et al. 1998; Quastel et al. 1997; Saiko et al. 1997; Vermiglio et al. 1999; Vykhovanets et al. 1997). A significant upward shift in thyroid- stimulating hormone (TSH) levels and increased rates of juvenile hypothyroidism in children exposed in Belarus and Ukraine because of the Chornobyl accident have been reported in some ecologic studies (Goldsmith et al. 1999; Quastel et al. 1997; Vykhovanets et al. 1997), but not in others (Kasatkina et al. 1997; Pacini et al. 1998; Saiko et al. 1997; Vermiglio et al. 1999). Several studies, including ours (Tronko et al. 2006a), suggested effects on thyroid autoimmunity (Pacini et al. 1998; Vermiglio et al. 1999; Vykhovanets et al. 1997). We have assessed the prevalence of autoimmune thyroiditis (AIT) and elevated antibodies to thyroid peroxidase (ATPO) in a cohort of 12,240 individuals from Ukraine who were < 18 years of age at the time of the Chornobyl accident, with individual thyroid radioactivity measurements taken shortly after the accident (Tronko et al. 2006a). We found a significant dose–response relationship for the prevalence of elevated ATPO alone, but not for AIT.

Here, we extend analyses in this cohort to test the hypothesis that the prevalence of hypothyroidism is associated with individual ^131^I thyroid dose estimates and/or that this relationship varies according to ATPO levels. Clarifying the relationship of hypothyroidism with radioiodines is important because of the widespread use of ^131^I in medical practice and concerns about health risks associated with the potential release of radioiodines from nuclear reactors.

## Materials and Methods

### Study population

**Figure 1:**
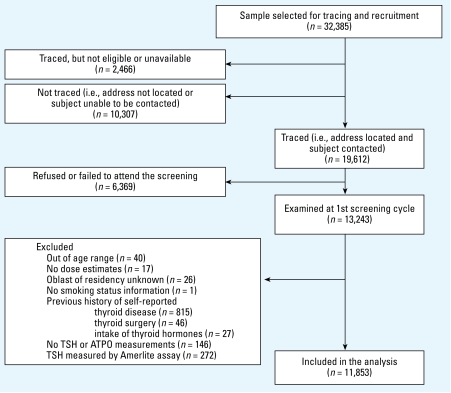
Study flow diagram.

A detailed description of the study design and methods was published previously (Stezhko et al. 2004). In brief, the cohort consists of individuals with direct measurements of thyroid radioactivity made in May or June 1986 who were < 18 years of age on the day of the accident (26 April 1986) and who resided in adjacent Chernihiv, Zhytomyr, or Kyiv oblasts (an oblast is an administrative area similar in size to a state or province) or in the city of Kyiv, Ukraine, at the beginning of the study (Stezhko et al. 2004). We based the present analysis on the data collected during the first screening examination conducted between 1998 and 2000. Figure 1 summarizes individuals we originally selected, traced, and screened (Stezhko et al. 2004), or excluded from the analysis. To assure that TSH measurements at first examination reflect spontaneous levels, we excluded from the analysis individuals who reported thyroid disease (*n* = 815, 90% with simple diffuse goiter) or thyroid surgery (*n* = 46) or intake of thyroid hormones (*n* = 27) before the first screening examination. We also excluded individuals without TSH or ATPO measurements (*n* = 146), mainly due to refusal to provide a serum sample, and those with measurements performed using the Amerlite assay at the beginning of the study before the Brahms assay became available (*n*= 272).

The study was reviewed and approved by the institutional review boards of the participating organizations in Ukraine and the United States, and all subjects gave written informed consent.

### Screening examination

Individuals were screened either by a mobile medical team visiting the local area or at the Research Institute of Endocrinology and Metabolism in Kyiv. Screening procedures included thyroid palpation and ultrasonographic examination by an ultrasonographer and independent clinical examination and palpation by an endocrinologist; a serum sample and a spot urine sample were collected. A series of structured questionnaires eliciting information on demographics, individual and family medical history, and items relevant to thyroid dose estimation, such as residential history, milk and leafy vegetable consumption, and iodine prophylaxis in May–June 1986, were administered by study personnel (Stezhko et al. 2004).

### Ultrasound examination

The thyroid was examined using a 7.5-MHz linear transducer (Toshiba 240; Toshiba Corp., Tokyo, Japan) with the subject supine and neck extended. The thyroid volume was calculated based on the volume of an ellipsoid (length × width × depth × 0.479) as described elsewhere (Brunn et al. 1981).

### Serum assays

We measured TSH, ATPO, and free thyroxine (FT_4_) in serum samples with LUMitest immunochemiluminescence assays (Brahms Diagnostica GMBH, Heningsdorf, Germany) using a Berthold 953 luminometer (Berthold Technologies, GmbH & Co. KG, Bad Wildbad, Germany). We performed TSH and ATPO measurements for everyone with a sufficient serum sample (99% of the cohort), and performed FT_4_ measurements only for those whose TSH result was outside the reference range. We conducted all assays according to the manufacturer’s instructions.

Intraassay coefficients of variation (CVs) for the TSH assay at 0.03 and 2.0 mIU/L were 3.0% and 2.2%, respectively, and the interassay CVs were 10.9% and 2.8%, respectively. Intraassay CVs for the ATPO assay at 84 and 375 U/mL were 8.1% and 6.5%, respectively, and the interassay CVs were 11.4% and 7.7%, respectively. Intraassay CVs for the FT_4_ assay at 7.4 and 33.5 pmol/L were 5.6% and 2.8%, respectively, and the inter-assay CVs at 6.7 and 53.9 pmol/L were 9.0% and 7.3%, respectively.

Based on evaluation of the range of values in a sample from our cohort, we set reference limits for TSH between 0.3 and 4.0 mIU/L. We considered ATPO > 60 U/mL to be elevated or positive, consistent with Brahms. We set reference limits for FT_4_ between 10.0 and 25.0 pmol/L based on Brahms’s recommendation.

### Iodine determination

We measured urinary iodine content, expressed in micrograms per liter, in spot urine samples using the Sandell–Kolthoff reaction (Tronko et al. 2005). The analytic sensitivity of the assay was 6 μg/L.

### Outcome definition

We defined hypothyroidism as serum TSH concentration > 4.0 mIU/L, the upper limit of the reference range. We limited overt hypothyroidism to cases of hypothyroidism with serum FT4 concentration < 10 pmol/L, the lower limit of the reference range.

### Dosimetry

Details of dosimetric methods have been published elsewhere (Likhtarev et al. 2003, 2006; Likhtarov et al. 2005, 2006). The unique feature of our study is that doses were primarily based on thyroid radioactivity measurements taken within 8 weeks of the accident. Using these measurements, data on dietary and lifestyle habits, and environmental transfer models, we estimated individual ^131^I thyroid doses and their uncertainties. The distributions of 1,000 individual thyroid dose estimates, obtained using a Monte Carlo procedure, were approximately lognormal (Likhtarev et al. 2003). For the present analyses, we used the arithmetic means of the 1,000 individual ^131^I dose realizations after adjustment for the typical thyroid masses of the Ukrainian population, which were measured by the Sasakawa Memorial Health Foundation for children 5–16 years of age and by the Ukrainian Radiation Protection Institute for children < 5 years of age (Likhtarov I, personal communication). The arithmetic mean and the median of the individual ^131^I arithmetic means in the cohort were 0.79 and 0.26 Gy, respectively. The dose estimates are for ^131^I, which typically accounts for more than 90% of the total thyroid dose (Stezhko et al. 2004). We did not take into account the remaining portions of thyroid dose from external exposure and from internal exposure to cesium and other isotopes of iodine.

### Statistical analysis

To estimate odds ratios (ORs) and compute the corresponding 95% confidence intervals (CIs), we conducted logistic regression analyses using the GMBO module of Epicure, version 1.8, for Microsoft Windows (Preston et al. 1993). We assumed that the hypothyroidism prevalence, γ(*x*, *d*), depends on a vector of covariates *x* that describes the background prevalence (i.e., in the absence of ^131^I exposure), and the estimated ^131^I thyroid dose *d*. Components of *x* or adjustment factors included indicator variables for categories of sex, age at examination, oblast of residency at the time of screening, current smoking status, vitamin consumption, history of thyroid disease in relatives, calendar year and season of examination, serum ATPO, and urinary iodine level. Under a relative OR model, γ can be written as a product of the background hypothyroidism odds, denoted γ_0_(*x*), and a dose–response function, *h*(*d*). If the dose–response function, *h*, is linear in dose alone, the model is γ(*x*, *d*) = γ_0_(*x*) × (1 + β*d*), where β is the excess odds ratio (EOR), the parameter that measures the unit increase in EOR per unit increase in dose in grays (EOR/Gy). We evaluated deviations from the linear model by fitting the linear-quadratic and linear-exponential dose–response models, γ(*x*, *d*) = γ_0_(*x*) × (1 + β*d* + θ*d*^2^) and γ(*x*, *d*) = γ_0_(*x*) × (1 + β*d*)*e^−^^θ^*^d^*, respectively, where θ, is a parameter that measures nonlinearity. We evaluated the statistical significance of model parameters, test of trend, and comparison in goodness of fit between the models using likelihood ratio chi-square test with degrees of freedom (df) equal to the difference in number of parameters in compared models. All statistical tests were two-sided, and we considered *p*< 0.05 significant.

To evaluate interaction, we allowed the dose–response trend, β, to vary within *J* categories of different factors such as sex, age at exposure, and ATPO, and compared deviance of the simple EOR model with a model that had *J* dose–response parameters. Under the null hypothesis of no interaction, the difference in model deviances asymptotically had a chi-square distribution with *J* − 1 df. A significant *p*-value indicated that the effect of radiation on hypothyroidism prevalence is not homogeneous across levels of the factor under investigation.

To investigate a potential bias from excluding the large number of subjects with prior self-reported thyroid diseases, we repeated analyses including these individuals.

## Results

Among 11,853 individuals included in the analysis, 719 (6.1%) had TSH levels > 4 mIU/L and 22 (0.2%) had TSH levels > 10 mIU/L. Among individuals with elevated TSH levels, 14 (1.9%) had overt hypothyroidism. Excluding cases with overt hypothyroidism had no effect on the study results, so we retained these cases in the analysis.

The analysis cohort was 49% female. At the time of the accident, the mean ± SD age of cohort members was 8 ± 4.7 years, with a mean age at examination (1998–2000) of 21.6 ± 4.9 years. The mean (median) ^131^I thyroid dose was 0.79 (0.26) Gy.

### Background prevalence of hypothyroidism

Table 1 summarizes selected associations for background prevalence of hypothyroidism. The prevalence of hypothyroidism was somewhat lower in females than males (*p* = 0.07). Residents of Kyiv and Chernihiv oblasts had 20% lower risk compared with residents of Zhytomyr Oblast (*p* = 0.02). We found no difference in prevalence between rural and urban residents (*p* = 0.48, not shown). Current smokers had lower risk of hypothyroidism prevalence compared with nonsmokers (*p* < 0.001). Individuals who reported intake of multi-vitamins had lower risk of hypothyroidism than did those who did not (*p* = 0.04). Presence of any thyroid disease in at least one relative somewhat increased risk of hypothyroidism (*p* = 0.12). Individuals examined in 1998 or 2000 had lower risk of hypothyroidism than those examined in 1999 (*p* < 0.001). There was a suggestion that risk of hypothyroidism was lower among individuals examined in spring or summer compared with those examined in the winter (*p* = 0.08). Individuals with ATPO > 60 U/mL had almost twice the risk of hypothyroidism compared with those with ATPO ≤ 60 U/mL (*p* = 0.001). We found no evidence that the risk of hypothyroidism varied with iodine intake, using several indicators: levels of urinary iodine (*p* = 0.89), presence of diffuse goiter (*p* = 0.56; data not shown), or large thyroid volume (≥ 15 mL) at ultrasound (*p* = 0.20; data not shown).

Table 2 shows associations for background prevalence of hypothyroidism with age at examination, by sex. Although the risk of hypothyroidism decreased with age for both sexes, the decrease was more pronounced and gradual in males than in females (*p* < 0.001).

### Radiation effects

**Figure 2:**
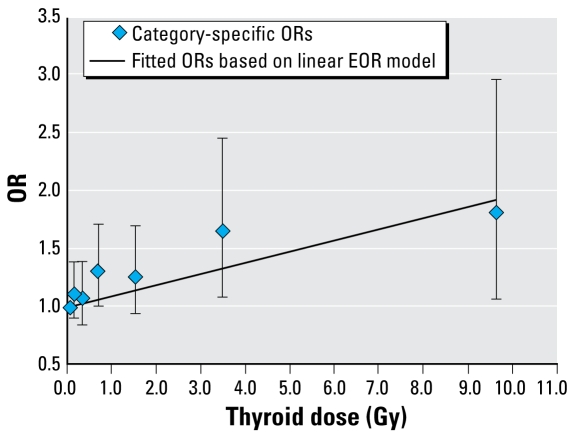
Association between prevalence of subclinical hypothyroidism and ^131^I thyroid dose estimates: Ukrainian–American cohort study of thyroid cancer and other thyroid diseases after the Chornobyl accident, 1998–2000. Dose–response line was adjusted to pass through the lowest ^131^I dose category. Data points are ^131^I dose category-specific ORs with 95% CIs. Line represents fitted ORs based on linear EOR model.

Table 3 shows adjusted thyroid dose category-specific ORs for hypothyroidism. We chose these categories to assure reasonably even proportional increment in ^131^I dose. The ORs increased with dose over the entire range (*p* = 0.004). Based on the simple linear EOR model, we found a highly significant dose–response (*p* = 0.003) with an estimated EOR/Gy of 0.10 (95% CI, 0.03– to 0.21). Figure 2 presents the category-specific ORs and fitted linear dose response. We found little evidence of nonlinearity in the dose response when we compared the linear fit with a linear-quadratic model (*p* = 0.89) or a linear-exponential model (*p* = 0.87). The linear dose–response trend was significant at doses < 5 Gy, with an estimated EOR/Gy of 0.16 (95% CI, 0.02 to 0.34), and there were positive, although not significant, linear trends at dose ranges < 3 Gy and < 2 Gy with estimated EORs/Gy of 0.13 (95% CI, −0.04 to 0.36) and 0.15 (95% CI, −0.07 to 0.45), respectively. If we defined subclinical hypothyroidism as TSH > 3.0 mIU/L, the magnitude of the dose response was similar, with an estimated EOR/Gy of 0.08 (95% CI, 0.03 to 0.15) over the entire dose range. Similarly, when we repeated the dose–response analyses including individuals with a history of thyroid disease or intake of thyroid hormones before the first screening, the EOR/Gy was 0.09 (95% CI, 0.03 to 0.17).

Table 4 summarizes variation in the dose–response slope according to selected characteristics. EOR per gray did not vary significantly by sex (*p* = 0.36), age at exposure (*p* = 0.56), current smoking status (*p* = 0.46), family history of thyroid disease (*p* = 0.79; data not shown), oblast of residency (*p* = 0.16; data not shown), year of examination (*p* = 0.83; data not shown), or levels of urinary iodine (*p* = 0.92). We observed highly significant variation of the dose–response according to ATPO levels (*p* < 0.001). Individuals with ATPO ≤ 60 U/mL had a higher EOR per gray compared with individuals with ATPO > 60 U/mL, for whom the EOR per gray did not significantly differ from zero, as evidenced by CIs.

## Discussion

We found a significant linear dose–response relationship between low to moderate ^131^I doses to the thyroid and prevalence of subclinical hypothyroidism among individuals who were children or adolescents at the time of the Chornobyl accident and underwent an in-depth thyroid examination 12–14 years later. After controlling for a variety of potential confounders, the overall estimated EOR per gray was 0.10 (95% CI, 0.03 to 0.21). EOR per gray was higher in individuals with ATPO levels ≤ 60 U/mL compared with those with ATPO > 60 U/mL.

We do not believe that the results of our study could be attributed to selection bias, even though we screened 40.9% of those selected from the file of thyroid activity measurements, representing 67.5% of those invited to participate, because distribution of measured thyroid radioactivity was similar among participants (*n* = 13,243) and nonparticipants (*n* = 19,142) (Stezhko et al. 2004). Further, we adjusted our analyses for a variety of possible confounders and effect modifiers to avoid any potential effects of differential distribution of such variables among participants and non-participants. Additional exclusion of individuals with self-reported history of thyroid disease or intake of thyroid hormones before the first screening is unlikely to have introduced bias because the results of analyses including and excluding these individuals were similar.

We did not take into account the impact of uncertainties in dose estimates in our analysis aimed at providing initial evidence on the relationship between prevalent hypothyroidism and environmental ^131^I exposure. We previously reported that uncertainties in estimates of the individual ^131^I thyroid doses were approximately lognormally distributed, with geometric standard deviations ranging from 1.6 to 5.0 and a median of 1.7 (Likhtarev et al. 2003). These uncertainties are comparable with, and in most cases smaller than, those reported in other studies of environmental ^131^I exposure (Davis et al. 2004; Lyon et al. 2006), primarily because all the participants had individual thyroid radioactivity measurements taken shortly after the accident. Two sources of uncertainty in thyroid dose estimates in our study, accounting for > 95% of overall uncertainty, were thyroid mass of the subject at the time of the measurement and measured radioactivity in thyroid (Likhtarev et al. 2003). Because the error for thyroid mass is likely to be a mixture of classical and Berkson error, and the error for direct thyroid measurement is largely classical, our study should have a mixed error structure. Using data from a study of thyroid disease in relation to radiation fallout from the Nevada test site, Mallick et al. (2002) showed that accounting for measurement error, based on a mixture error model, resulted in radiation risk estimates that were higher than unadjusted estimates, but < 100% higher when all error was assumed to be classical. Based on these results, it seems reasonable to assume that the true radiation risk for hypothyroidism is likely to be higher than what we report here, although with greater uncertainty.

It is well known that high thyroid doses received during therapeutic external irradiation of the head and neck for childhood cancer (ranging from 30 to 70 Gy) and from ^131^I radiotherapy for Graves disease (typically ≥ 50 Gy) result in direct killing of thyroid cells and lead to subsequent development of structural and/or functional abnormalities of the thyroid, with primary hypothyroidism being the most common thyroid dysfunction (Cooper 2005; Hancock et al. 1995; Jereczek-Fossa et al. 2004). By contrast, data concerning risk of hypothyroidism after diagnostic ^131^I administration and environmental exposure due to nuclear weapons tests and releases from nuclear reactors have been limited and inconsistent (Davis et al. 2004; Larsen et al. 1982; Lyon et al. 2006; Takahashi et al. 1999).

Our finding of increased prevalence of subclinical hypothyroidism after ^131^I exposure is in agreement with earlier observations (Larsen et al. 1982) but not later ones (Takahashi et al. 1999) in Marshall Islanders exposed to ^131^I at doses from 1.35 to 3.35 Gy as a consequence of nuclear weapons testing. The latter negative finding for the Marshallese is consistent with the results of the Hanford Thyroid Disease Study, which 50 years after exposures from the Hanford nuclear facility (Washington State, USA) found no association between hypothyroidism, based on various outcome definitions, and ^131^I dose estimates [mean (median) thyroid dose, 0.20 (0.10) Gy] (Davis et al. 2004). Reevaluation of thyroid disease risk in relation to exposure from the Nevada nuclear test site suggested that risk of AIT with hypothyroidism may increase with thyroid dose (*p* = 0.18), but this finding was based on only 35 cases (Lyon et al. 2006). The data from atomic bomb survivors exposed to external high-dose-rate gamma irradiation in a range from 0 to 4 Gy are also inconsistent (Imaizumi et al. 2006; Nagataki et al. 1994). Although in an earlier study a significant bell-shaped dose–response curve for antibody-positive hypothyroidism was reported (Nagataki et al. 1994), it was not confirmed in a later study (Imaizumi et al. 2006). Inconsistent findings in all these studies may reflect the unique circumstances of population exposure, including different types of radiation or mix of radionuclides, dose rates, age at exposure, or time since exposure, assuming that radiation risk for hypothyroidism varies by these factors.

Comparison of our study with others conducted in populations exposed to the Chornobyl fallout during childhood is generally difficult because previous research lacked individual dose estimates. In several studies conducted in Belarus and Ukraine, a significant upward shift in TSH levels and increased rates of juvenile hypothyroidism were reported in children living in radionuclide-contaminated areas compared with children from uncontaminated areas (Goldsmith et al. 1999; Quastel et al. 1997; Vykhovanets et al. 1997). The remaining ecologic studies were essentially negative (Kasatkina et al. 1997; Pacini et al. 1998; Saiko et al. 1997; Vermiglio et al. 1999).

The observed association between the prevalence of subclinical hypothyroidism and ^131^I dose in our study, although highly significant (*p* = 0.003), is small, with an estimated 0.10 EOR/Gy. Nevertheless, this association appears to be robust and unlikely to be attributable to confounding because adjustment for a variety of important factors had no meaningful effect on the estimate. We found little evidence of a departure of the dose response from linearity based on several alternative models. When we excluded from the analysis individuals with doses ≥ 2 Gy, the EOR per gray, although not statistically significant, did not change materially (EOR/Gy = 0.15), supporting linearity of the dose response even at lower dose levels. If the true relationship between hypothyroidism prevalence and ^131^I thyroid dose is close to what we observed, most previous studies would have had low statistical power to detect a relationship.

After controlling for the effect of ^131^I, the relationships with other risk factors for hypothyroidism in our study were similar to those reported in nonirradiated populations: a doubling in risk with elevated ATPO levels (Bjøro et al. 2000; Hollowell et al. 2002; Hoogendoorn et al. 2006; Knudsen et al. 1999; O’Leary et al. 2006; Pedersen et al. 2003; Vanderpump et al. 1995), somewhat increased risk with family history of thyroid disease (Surks et al. 2004), decreased risk with current smoking (Åsvold et al. 2007; Belin et al. 2004; Jorde and Sundsfjord 2006), decreased risk with intake of vitamins (Zimmermann et al. 2004), and variation in risk according to geographical area (Flynn et al. 2004) and season of blood draw (Andersen et al. 2003). The age-related decrease in risk of background prevalence of hypothyroidism for both sexes was also in agreement with the decline reported in other populations of comparable age (Marwaha et al. 2008; Zurakowski et al. 1999).

The relationship among serum ATPO, ^131^I, and risk of hypothyroidism in our study was complex. When included in the same model simultaneously, the associations of risk with ^131^I and ATPO were comparable with the respective associations when these factors were included individually. This observation does not support the idea that the radiation-related increase in prevalence of subclinical hypothyroidism could be attributed to the radiation-related increase in prevalence of ATPO that we reported previously (Tronko et al. 2006a). In fact, we found a significant interaction between ^131^I and ATPO level in the prevalence of subclinical hypothyroidism, suggesting that the effects of ^131^I and ATPO were not statistically independent. Specifically, individuals with ATPO ≤ 60 U/mL had a stronger dose–response relationship than did individuals with ATPO > 60 U/mL. Today, little is known about biological and clinical consequences of low doses of ^131^I on the thyroid gland and whether they cause sufficient direct or indirect thyroid damage that could result in subclinical hypothyroidism. It has been hypothesized that the ^131^I-related increase, at least for ATPO antibodies, may be transitory (Agate et al. 2008). Given the fact that our analysis is cross-sectional in nature and that findings from clinical studies concerning the role of antibodies in the development of hypothyroidism after ^131^I therapy are conflicting (Ceccarelli et al. 2005; Chiovato et al. 1998; Mariotti et al. 1986), prospective data are needed to characterize the dynamic of radiation-related risk of hypothyroidism and to confirm its relation with ATPO.

In summary, this is the first study to find a significant relationship between prevalence of hypothyroidism and individual ^131^I thyroid doses due to environmental exposure. The radiation effect was small, a 10% increase per gray, and was largely limited to subclinical hypothyroidism. Additional data from prospective studies is required to enhance our understanding of the long-term consequences of ^131^I exposure on thyroid function and to clarify what role antithyroid antibodies have in this process.

## Figures and Tables

**Table 1:** ORs and 95% CIs for background prevalence of hypothyroidism (TSH > 4 mIU/L) among individuals exposed to ^131^I from the Chornobyl accident.

Parameter	Cases (*n* = 719)	Noncases (*n* = 11,134)	Rate per 1,000	OR (95% CI)^a^
Sex				

Male	363	5,723	59.64	1.00 (Referent)
Female	356	5,411	61.73	0.86 (0.58–1.28)
*p*-Valuehomogeneity: 0.07, df = 1				

Place of residency				

Zhytomyr Oblast	197	3,044	60.78	1.00 (Referent)
Kyiv Oblast/Kyiv City	123	2,269	51.42	0.80 (0.62–1.03)
Chernihiv Oblast	399	5,821	61.15	0.78 (0.58–0.87)
*p*-Value homogeneity: 0.02, df = 2				

Current smoking				

No	536	7,343	68.03	1.00 (Referent)
Yes	183	3,791	46.05	0.71 (0.58–0.87)
*p*-Value homogeneity: < 0.001, df = 1				

Vitamin consumption				

No	690	10,477	61.79	1.00 (Referent)
Yes	29	657	42.27	0.68 (0.45–0.98)
*p*-Value homogeneity: 0.04, df = 1				

Thyroid disease history in relatives				

No	486	7,522	60.69	1.00 (Referent)
At least one relative affected	71	811	80.50	1.24 (0.94–1.61)
*p*-Value homogeneity: 0.12, df = 1				
Unknown	162	2,801	54.67	0.88 (0.73–1.06)

Year of examination				

1998	23	1,284	17.60	0.12 (0.07–0.19)
1999	244	2,577	86.49	1.00 (Referent)
2000	452	7,273	58.51	0.80 (0.67–0.96)
*p*-Value homogeneity: < 0.001, df = 2				

Season of examination				

December–February	154	1,950	73.19	1.00 (Referent)
March–May	232	3,195	67.70	0.81 (0.64–1.02)
June–August	150	2,722	52.23	0.74 (0.58–0.94)
September–November	183	3,267	53.04	0.90 (0.71–1.15)
*p*-Value homogeneity: 0.08, df = 3				

ATPO level (U/mL)				

≤ 60	591	10,468	56.46	1.00 (Referent)
> 60	128	1,385	95.31	1.81 (1.47–2.23)
*p*-Value homogeneity: < 0.001, df = 1				

Urinary iodine (μg/L)				

< 20	101	1,428	66.06	1.08 (0.83–1.40)
20–49	284	4,321	61.67	1.03 (0.85–1.25)
50–99	191	3,093	58.16	1.00 (Referent)
≥ 100	73	1,262	54.68	0.95 (0.72–1.26)
*p*-Value homogeneity: 0.89, df = 3				
Missing/unknown	70	1,030	63.64	1.10 (0.83–1.48)

a All analyses performed with adjustment for sex, age at examination by sex, place of residency, current smoking status, vitamin consumption, family history of thyroid disease, year and season of examination, ATPO level, and ^131^I thyroid dose based on linear EOR model.

**Table 2:** Sex- and age-specific ORs and 95% CIs for background prevalence of hypothyroidism (TSH > 4 mIU/L) among individuals exposed to ^131^I from the Chornobyl accident.

	Male				Female			
Age at examination (years)	Cases (*n*)	Noncases (*n*)	Rate per 1,000	OR (95% CI)^a^	Cases (*n*)	Noncases (*n*)	Rate per 1,000	OR (95% CI)^a^
12–14	59	566	94.40	1.0 (Referent)	54	567	86.96	1.0 (Referent)
15–19	143	1,623	80.97	0.83 (0.59–1.17)	106	1,639	60.74	0.60 (0.42–0.86)
20–24	83	1,802	44.03	0.46 (0.31–0.67)	95	1,662	54.07	0.53 (0.36–0.76)
25–32	78	1,732	43.09	0.43 (0.29–0.64)	101	1,543	61.43	0.59 (0.40–0.85)
*p*-Value homogeneity	< 0.001, df = 3				0.01, df = 3			

a Analysis performed with adjustment for place of residency, current smoking status, vitamin consumption, family history of thyroid disease, year and season of examination, ATPO level, and ^131^I thyroid dose based on linear EOR model.

**Table 3:** ORs and 95% CIs with thyroid dose for prevalence of hypothyroidism (TSH > 4 mIU/L) among individuals exposed to ^131^I from the Chornobyl accident.

Thyroid dose range (Gy)	Mean dose (Gy)	Cases (*n* = 719)	Noncases (*n* = 11,134)	Rate per 1,000	OR^a^ (95% CI)
0.00–0.099	0.05	182	3,571	48.49	1.00 (Referent)
0.10–0.249	0.16	167	2,620	59.92	1.11 (0.89–1.39)
0.25–0.49	0.35	121	1,878	60.53	1.08 (0.83–1.39)
0.50–0.99	0.71	105	1,349	72.21	1.31 (1.00–1.72)
1.00–2.49	1.54	86	1,153	69.41	1.27 (0.94–1.71)
2.50–4.99	3.49	36	370	88.67	1.67 (1.09–2.49)
≥ 5.00	9.63	22	193	102.32	1.83 (1.07–3.00)
*p*-Value for trend = 0.004, df = 1					

a After adjustment for effects of sex, age at examination by sex, oblast of residency, current smoking status, vitamin consumption, history of thyroid disease in relatives, year and season of examination, and ATPO level.

**Table 4:** EORs/Gy and 95% CIs for prevalence of hypothyroidism (TSH > 4 mIU/L) according to selected characteristics among individuals exposed to ^131^I because of the Chornobyl accident.

Characteristic	EOR/Gy (95% CI)^a^
Sex	

Male	0.14 (0.03 to 0.33)
Female	0.07 (−0.01 to 0.19)
*p*-Value homogeneity^b^	0.36, df = 1

Age at exposure, years	

< 1	0.11 (0.01 to 0.32)
1–4	0.06 (−0.02 to 0.19)
5–9	0.12 (−0.03 to 0.36)
10–14	0.11 (−0.06 to 0.41)
15–18	0.51 (0.01 to 1.42)
*p*-Value homogeneity^b^	0.56, df = 4

Current smoking status	

No	0.08 (0.01 to 0.20)
Yes	0.17 (0.002 to 0.47)
*p*-Value homogeneity^b^	0.46, df = 1

Urinary iodine (μg/L)	

< 20	0.11 (−0.03 to 0.35)
20–49	0.09 (0.01 to 0.22)
50–99	0.12 (0.001 to 0.31)
≥ 100	0.003 (−0.15 to 0.35)
Unknown	0.18 (−0.07 to 0.63)
*p*-Value homogeneity^b^	0.92, df = 4

ATPO level (U/mL)	

≤ 60	0.16 (0.13 to 0.20)
> 60	−0.14 (−0.22 to 0.15)
*p*-Value homogeneity^b^	< 0.001, df = 1

a Adjusted for effects of sex, age at examination by sex, oblast of residency, current smoking status, vitamin consumption, history of thyroid disease in relatives, year and season of examination, and ATPO level.

b *p*-Value based on maximum likelihood ratio test.

## References

[b1-ehp-117-745] Agate L, Mariotti S, Elisei R, Mossa P, Pacini F, Molinaro E (2008). Thyroid autoantibodies and thyroid function in subjects exposed to Chernobyl fallout during childhood: evidence for a transient radiation-induced elevation of serum thyroid antibodies without an increase in thyroid autoimmune disease. J Clin Endocrinol Metab.

[b2-ehp-117-745] Andersen S, Bruun NH, Pedersen KM, Laurberg P (2003). Biologic variation is important for interpretation of thyroid function tests. Thyroid.

[b3-ehp-117-745] Åsvold BO, Bjøro T, Nilsen TI, Vatten LJ (2007). Tobacco smoking and thyroid function: a population-based study. Arch Intern Med.

[b4-ehp-117-745] Belin RM, Astor BC, Powe NR, Ladenson PW (2004). Smoke exposure is associated with a lower prevalence of serum thyroid autoantibodies and thyrotropin concentration elevation and a higher prevalence of mild thyrotropin concentration suppression in the third National Health and Nutrition Examination Survey (NHANES III). J Clin Endocrinol Metab.

[b5-ehp-117-745] Bjøro T, Holmen J, Krüger Ø, Midthjell K, Hunstad K, Schreiner T (2000). Prevalence of thyroid disease, thyroid dysfunction and thyroid peroxidase antibodies in a large, unselected population. The Health Study of Nord-Trøndelag (HUNT). Eur J Endocrinol.

[b6-ehp-117-745] Brunn J, Block U, Ruf G, Bos I, Kunze WP, Scriba PC (1981). Volumetric analysis of thyroid lobes by real-time ultrasound. Dtsch Med Wochenschr.

[b7-ehp-117-745] Cardis E, Kesminiene A, Ivanov V, Malakhova I, Shibata Y, Khrouch V (2005). Risk of thyroid cancer after exposure to ^131^I in childhood. J Natl Cancer Inst.

[b8-ehp-117-745] Ceccarelli C, Bencivelli W, Vitti P, Grasso L, Pinchera A (2005). Outcome of radioiodine-131 therapy in hyperfunctioning thyroid nodules: a 20 years’ retrospective study. Clin Endocrinol (Oxf).

[b9-ehp-117-745] Chiovato L, Fiore E, Vitti P, Rocchi R, Rago T, Dokic D (1998). Outcome of thyroid function in Graves’ patients treated with radioiodine: role of thyroid-stimulating and thyroid-blocking antibodies and of radioiodine-induced thyroid damage. J Clin Endocrinol Metab.

[b10-ehp-117-745] Cooper DS, Braverman LE, Utiger RD (2005). Treatment of thyrotoxicosis. Werner and Ingbar’s The Thyroid: A Fundamental and Clinical Text.

[b11-ehp-117-745] Davis S, Kopecky KJ, Hamilton TE, Onstad L, Hanford Thyroid Study Team (2004). Thyroid neoplasia, autoimmune thyroiditis, and hypothyroidism in persons exposed to iodine 131 from the Hanford nuclear site. JAMA.

[b12-ehp-117-745] Eheman CR, Garbe P, Tuttle RM (2003). Autoimmune thyroid disease associated with environmental thyroidal irradiation. Thyroid.

[b13-ehp-117-745] Flynn RW, MacDonald TM, Morris AD, Jung RT, Leese GP (2004). The thyroid epidemiology, audit, and research study: thyroid dysfunction in the general population. J Clin Endocrinol Metab.

[b14-ehp-117-745] Goldsmith JR, Grossman CM, Morton WE, Nussbaum RH, Kordysh EA, Quastel MR (1999). Juvenile hypothyroidism among two populations exposed to radioiodine. Environ Health Perspect.

[b15-ehp-117-745] Hancock SL, McDougall IR, Constine LS (1995). Thyroid abnormalities after therapeutic external radiation. Int J Radiat Oncol Biol Phys.

[b16-ehp-117-745] Hollowell JG, Staehling NW, Flanders WD, Hannon WH, Gunter EW, Spencer CA (2002). Serum TSH, T(4), and thyroid antibodies in the United States population (1988 to 1994): National Health and Nutrition Examination Survey (NHANES III). J Clin Endocrinol Metab.

[b17-ehp-117-745] Hoogendoorn EH, Hermus AR, de Vegt F, Ross HA, Verbeek AL, Kiemeney LA (2006). Thyroid function and prevalence of anti-thyroperoxidase antibodies in a population with borderline sufficient iodine intake: influences of age and sex. Clin Chem.

[b18-ehp-117-745] Imaizumi M, Usa T, Tominaga T, Neriishi K, Akahoshi M, Nakashima E (2006). Radiation dose–response relationships for thyroid nodules and autoimmune thyroid diseases in Hiroshima and Nagasaki atomic bomb survivors 55–58 years after radiation exposure. JAMA.

[b19-ehp-117-745] Ivanov VK, Gorski AI, Tsyb AF, Maksioutov MA, Tumanov KA, Vlasov OK (2006). Radiation-epidemiological studies of thyroid cancer incidence among children and adolescents in the Bryansk oblast of Russia after the Chernobyl accident (1991–2001 follow-up period). Radiat Environ Biophys.

[b20-ehp-117-745] Jacob P, Kenigsberg Y, Zvonova I, Goulko G, Buglova E, Heidenreich WF (1999). Childhood exposure due to the Chernobyl accident and thyroid cancer risk in contaminated areas of Belarus and Russia. Br J Cancer.

[b21-ehp-117-745] Jereczek-Fossa BA, Alterio D, Jassem J, Gibelli B, Tradati N, Orecchia R (2004). Radiotherapy-induced thyroid disorders. Cancer Treat Rev.

[b22-ehp-117-745] Jorde R, Sundsfjord J (2006). Serum TSH levels in smokers and non-smokers. The 5th Tromsø Study. Exp Clin Endocrinol Diabetes.

[b23-ehp-117-745] Kasatkina EP, Shilin DE, Rosenbloom AL, Pykov MI, Ibragimova GV, Sokolovskaya VN (1997). Effects of low level radiation from the Chernobyl accident in a population with iodine deficiency. Eur J Pediatr.

[b24-ehp-117-745] Knudsen N, Jørgensen T, Rasmussen S, Christiansen E, Perrild H (1999). The prevalence of thyroid dysfunction in a population with borderline iodine deficiency. Clin Endocrinol (Oxf).

[b25-ehp-117-745] Larsen PR, Conard RA, Knudsen KD, Robbins J, Wolff J, Rall JE (1982). Thyroid hypofunction after exposure to fallout from a hydrogen bomb explosion. JAMA.

[b26-ehp-117-745] Likhtarev I, Bouville A, Kovgan L, Luckyanov N, Voillequé P, Chepurny M (2006). Questionnaire- and measurement-based individual thyroid doses in Ukraine resulting from the Chornobyl nuclear reactor accident. Radiat Res.

[b27-ehp-117-745] Likhtarev I, Minenko V, Khrouch V, Bouville A (2003). Uncertainties in thyroid dose reconstruction after Chernobyl. Radiat Prot Dosimetry.

[b28-ehp-117-745] Likhtarov I, Kovgan L, Vavilov S, Chepurny M, Bouville A, Luckyanov N (2005). Post-Chornobyl thyroid cancers in Ukraine. Report 1: estimation of thyroid doses. Radiat Res.

[b29-ehp-117-745] Likhtarov I, Kovgan L, Vavilov S, Chepurny M, Ron E, Lubin J (2006). Post-Chornobyl thyroid cancers in Ukraine. Report 2: risk analysis. Radiat Res.

[b30-ehp-117-745] Lyon JL, Alder SC, Stone MB, Scholl A, Reading JC, Holubkov R (2006). Thyroid disease associated with exposure to the Nevada nuclear weapons test site radiation. Epidemiology.

[b31-ehp-117-745] Mallick B, Hoffman FO, Carroll RJ (2002). Semiparametric regression modeling with mixtures of Berkson and classical error, with application to fallout from the Nevada test site. Biometrics.

[b32-ehp-117-745] Mariotti S, Martino E, Francesconi M, Ceccarelli C, Grasso L, Lippi F (1986). Serum thyroid autoantibodies as a risk factor for development hypothyroidism after radioactive iodine therapy for single thyroid “hot” nodule. Acta Endocrinol (Copenh).

[b33-ehp-117-745] Marwaha RK, Tandon N, Desai A, Kanwar R, Grewal K, Aggarwal R (2008). Reference range of thyroid hormones in normal Indian school-age children. Clin Endocrinol (Oxf).

[b34-ehp-117-745] Nagataki S, Shibata Y, Inoue S, Yokoyama N, Izumi M, Shimaoka K (1994). Thyroid diseases among atomic bomb survivors in Nagasaki. JAMA.

[b35-ehp-117-745] O’Leary PC, Feddema PH, Michelangeli VP, Leedman PJ, Chew GT, Knuiman M (2006). Investigations of thyroid hormones and antibodies based on a community health survey: the Busselton thyroid study. Clin Endocrinol (Oxf).

[b36-ehp-117-745] Pacini F, Vorontsova T, Molinaro E, Kuchinskaya E, Agate L, Shavrova E (1998). Prevalence of thyroid autoantibodies in children and adolescents from Belarus exposed to the Chernobyl radioactive fallout. Lancet.

[b37-ehp-117-745] Pedersen IB, Knudsen N, Jørgensen T, Perrild H, Ovesen L, Laurberg P (2003). Thyroid peroxidase and thyroglobulin autoantibodies in a large survey of populations with mild and moderate iodine deficiency. Clin Endocrinol (Oxf).

[b38-ehp-117-745] Preston DL, Lubin JH, Pierce DA, McConney ME (1993). Epicure.

[b39-ehp-117-745] Quastel MR, Goldsmith JR, Mirkin L, Poljak S, Barki Y, Levy J, Gorodischer R (1997). Thyroid-stimulating hormone levels in children from Chernobyl. Environ Health Perspect.

[b40-ehp-117-745] Saiko AS, Goncharenko OE, Daniliuk VV, Panasyuk GD, Derzhtskaya NK, Cot VA, Yamashita S, Shibata Y (1997). Findings of the Chernobyl Sasakawa Health and Medical Cooperation Project: abnormal thyroid echogenity and autoimmune thyroid diseases around Chernobyl. Chernobyl: A Decade.

[b41-ehp-117-745] Stezhko VA, Buglova EE, Danilova LI, Drozd VM, Krysenko NA, Lesnikova NR (2004). A cohort study of thyroid cancer and other thyroid diseases after the Chornobyl accident: objectives, design and methods. Radiat Res.

[b42-ehp-117-745] Surks MI, Ortiz E, Daniels GH, Sawin CT, Col NF, Cobin RH (2004). Subclinical thyroid disease: scientific review and guidelines for diagnosis and management. JAMA.

[b43-ehp-117-745] Takahashi T, Fujimori K, Simon SL, Bechtner G, Edwards R, Trott KR (1999). Thyroid nodules, thyroid function and dietary iodine in the Marshal Islands. Int J Epidemiol.

[b44-ehp-117-745] Tronko MD, Brenner AV, Olijnyk VA, Robbins J, Epstein OV, McConnell RJ (2006a). Autoimmune thyroiditis and exposure to iodine 131 in the Ukrainian cohort study of thyroid cancer and other thyroid diseases after the Chornobyl accident: results from the first screening cycle (1998–2000). J Clin Endocrinol Metab.

[b45-ehp-117-745] Tronko MD, Howe GR, Bogdanova TI, Bouville AC, Epstein OV, Brill AB (2006b). A cohort study of thyroid cancer and other thyroid diseases after the Chornobyl accident: thyroid cancer in Ukraine detected during first screening. J Natl Cancer Inst.

[b46-ehp-117-745] Tronko M, Kravchenko V, Fink D, Hatch M, Turchin V, McConnell R (2005). Iodine excretion in regions of Ukraine affected by the Chornobyl accident: experience of the Ukrainian-American cohort study of thyroid cancer and other thyroid diseases. Thyroid.

[b47-ehp-117-745] United Nations Scientific Committee on the Effects of Atomic Radiation (2000). Sources and Effects of Ionizing Radiation.

[b48-ehp-117-745] Vanderpump MP, Tunbridge WM, French JM, Appleton D, Bates D, Clark F (1995). The incidence of thyroid disorders in the community: a twenty-year follow-up of the Whickham Survey. Clin Endocrinol (Oxf).

[b49-ehp-117-745] Vermiglio F, Castagna MG, Volnova E, Lo Presti VP, Moleti M, Violi MA (1999). Post-Chernobyl increased prevalence of humoral thyroid autoimmunity in children and adolescents from a moderately iodine-deficient area in Russia. Thyroid.

[b50-ehp-117-745] Vykhovanets EV, Chernyshov VP, Slukvin II, Antipkin YG, Vasyuk AN, Klimenko HF (1997). ^131^I dose-dependent thyroid autoimmune disorders in children living around Chernobyl. Clin Immunol Immunopathol.

[b51-ehp-117-745] Zimmermann MB, Wegmüller R, Zeder C, Chaouki N, Torresani T (2004). The effects of vitamin A deficiency and vitamin A supplementation on thyroid function in goitrous children. J Clin Endocrinol Metab.

[b52-ehp-117-745] Zurakowski D, Di Canzio J, Maizoub JA (1999). Pediatric reference intervals for serum thyroxine, triiodothyronine, thyrotropin and free thyroxine. Clin Chem.

